# X-ray Dark-field Radiography - *In-Vivo* Diagnosis of Lung Cancer in Mice

**DOI:** 10.1038/s41598-017-00489-x

**Published:** 2017-03-24

**Authors:** Kai Scherer, Andre Yaroshenko, Deniz Ali Bölükbas, Lukas B. Gromann, Katharina Hellbach, Felix G. Meinel, Margarita Braunagel, Jens von Berg, Oliver Eickelberg, Maximilian F. Reiser, Franz Pfeiffer, Silke Meiners, Julia Herzen

**Affiliations:** 1 Lehrstuhl für Biomedizinische Physik, Physik-Department & Institut für Medizintechnik, Technische Universität München, 85748 Garching, Germany; 2 Philips Medical Systems DMC GmbH, 22335 Hamburg, Germany; 3 Comprehensive Pneumology Center (CPC), University Hospital, Ludwig-Maximilians University, Helmholtz Zentrum München, Member of the German Center for Lung Research (DZL), 81377 Munich, Germany; 4 Institute for Clinical Radiology, Ludwig-Maximilians-University Hospital Munich, 81377 Munich, Germany; 5 Philips Research Laboratories, Philips Medical Systems, 22335 Hamburg, Germany

## Abstract

Accounting for about 1.5 million deaths annually, lung cancer is the prevailing cause of cancer deaths worldwide, mostly associated with long-term smoking effects. Numerous small-animal studies are performed currently in order to better understand the pathogenesis of the disease and to develop treatment strategies. Within this letter, we propose to exploit X-ray dark-field imaging as a novel diagnostic tool for the detection of lung cancer on projection radiographs. Here, we demonstrate in living mice bearing lung tumors, that X-ray dark-field radiography provides significantly improved lung tumor detection rates without increasing the number of false-positives, especially in the case of small and superimposed nodules, when compared to conventional absorption-based imaging. While this method still needs to be adapted to larger mammals and finally humans, the technique presented here can already serve as a valuable tool in evaluating novel lung cancer therapies, tested in mice and other small animal models.

## Introduction

With approximately 1.5 million deaths a year worldwide, lung cancer is the leading cause of cancer-induced deaths^1, 2^. Lung cancer is mostly diagnosed at an advanced stage with poor prognosis and has an average five-year survival rate of 15%^3^. The initial assessment of the lung in clinical routine is often done by conventional chest radiography, however, the sensitivity of detecting small nodules is particularly low. A recent study, for instance, demonstrated that the detection sensitivity drops below 50% for tumors with a mean size of 19 mm^4^. Further, the average size of missed lung nodules on a X-ray radiography is no less than 15 mm^5^. These results may explain why conventional x-ray chest radiography is not optimal as a mortality reducing screening program for (early) detection of lung cancer^6^.

In order to better understand the pathogenesis and develop treatment strategies numerous small-animal studies are currently being performed. In these studies, it is essential to be able to precisely detect and track the development of lung nodules *in vivo*. Thus, it is possible to assess the impact of therapy over time. Currently, micro-CT and histology are used as the tools of choice for the lung nodule tracking. While histopathology offers an excellent opportunity, it is not suitable for longitudinal studies. Micro-CT can help localize the tumors, but it has a disadvantage of delivering quite a substantial dose to the animal. Accumulation of the dose can therefore bias or even falsify the results of the study^7^.

To address the aforementioned shortcomings, we propose to utilize the recently developed technique called X-ray dark-field radiography for the improved *in-vivo* assessment of lung cancer^8, 9^.

X-ray dark-field is a signal that quantifies the small-angle scattering that occurs in the lung at the air:tissue interfaces. Previous simulation^10^ and experimental results^11–14^ have demonstrated that changes to the size and number of the alveoli can be precisely detected with this imaging technique. Thus, recent studies with *in vivo* mice demonstrated promising potential in detection of pulmonary disorders such as emphysema^15, 16^, and pulmonary fibrosis^17^, amongst others^18, 19^.

**Figure 1:**
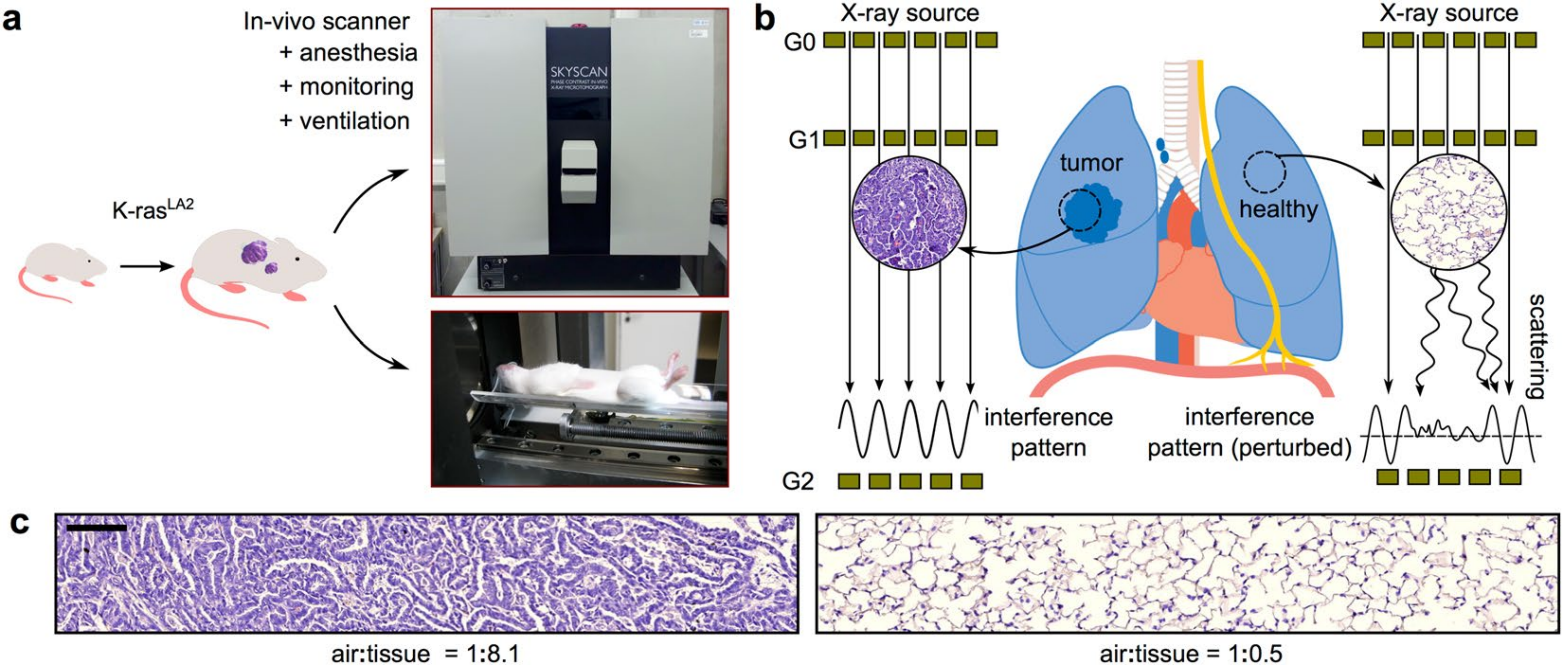
Study design and background of *in-vivo* small-animal dark-field imaging of lung cancer. (**a**) As a disease model, K-ras^LA2^ mice were used. These mice frequently exhibit high-grade adenocarcinomas, developing from early onset lung adenomas. *In-vivo* dark-field imaging was performed using the Skyscan small-animal scanner, which is based on a Talbot-Lau interferometer. (**b**) X-ray dark-field imaging is highly sensitive to distortions of the natural lung micro-anatomy, as indicated using typical histopathology images: a pronounced air-tissue interface in healthy lung tissue (right) causes heavy X-ray scattering. In cancerous tissue (left) the air:tissue volume ratio is reduced due to uncontrolled cell proliferation and loss of alveolar space and therefore X-ray scattering is strongly reduced. Subsequently, information on the scatter properties of the lung can be retrieved by evaluating a setup-specific interference pattern. (**c**) Magnified histological sections visualise the impact of cancer on tissue density and a change in air:tissue distribution, respectively. The scale bar corresponds to 100 μm. This figure contains a lung illustration adapted from https://commons.wikimedia.org/wiki/File:Diagram_1_of_3_showing_stage_3A_lung_cancer_CRUK_008.svg, which is licensed under the Attribution-ShareAlike 4.0 International license (http://creativecommons.org/licenses/by-sa/4.0/). Further a mouse sketch is adapted from https://commons.wikimedia.org/wiki/File:Knockout_mouse_breeding_scheme.svg, which licensed under the Attribution 3.0 Unported license (http://creativecommons.org/licenses/by/3.0/). Both licenses allow adaptation of images, even commercially.

Thereby, the study by Hellbach *et al.* ^16^ clearly revealed that the dark-field signal of the lung scales with the size of the alveoli, where in the publication by Yaroshenko *et al.* ^17^ it was demonstrated that the signal also decreases with the decreasing number of alveoli. Here we show, using an *in vivo* small-animal lung cancer model (Fig. 1a) that x-ray dark-field contrast is also highly suitable for the detection of small tumor nodules which could be overlooked at the corresponding conventional image, by monitoring the cancer-caused reduction of the air:tissue volume ratio (Fig. 1b and c)^20^.

Finally, our study is highly significant for both clinical imaging and treatment development, providing a diagnostic and dose-saving framework for the assessment and evaluation of new lung cancer therapeutics and their application in mouse models.

## Results

The detection of lung cancer using X-ray dark-field radiography was assessed in two steps. At first a qualitative radiographic investigation, using a transgenic animal model with spontaneous K-ras oncogene-driven growth of lung tumors which closely resembles human lung tumor development, was carried out. Subsequently, a quantitative reader study was conducted, with experienced radiologists to compare detection rates of lung tumor nodules with dark-field versus conventional radiographs.

### *In-vivo* dark-field radiography

**Figure 2:**
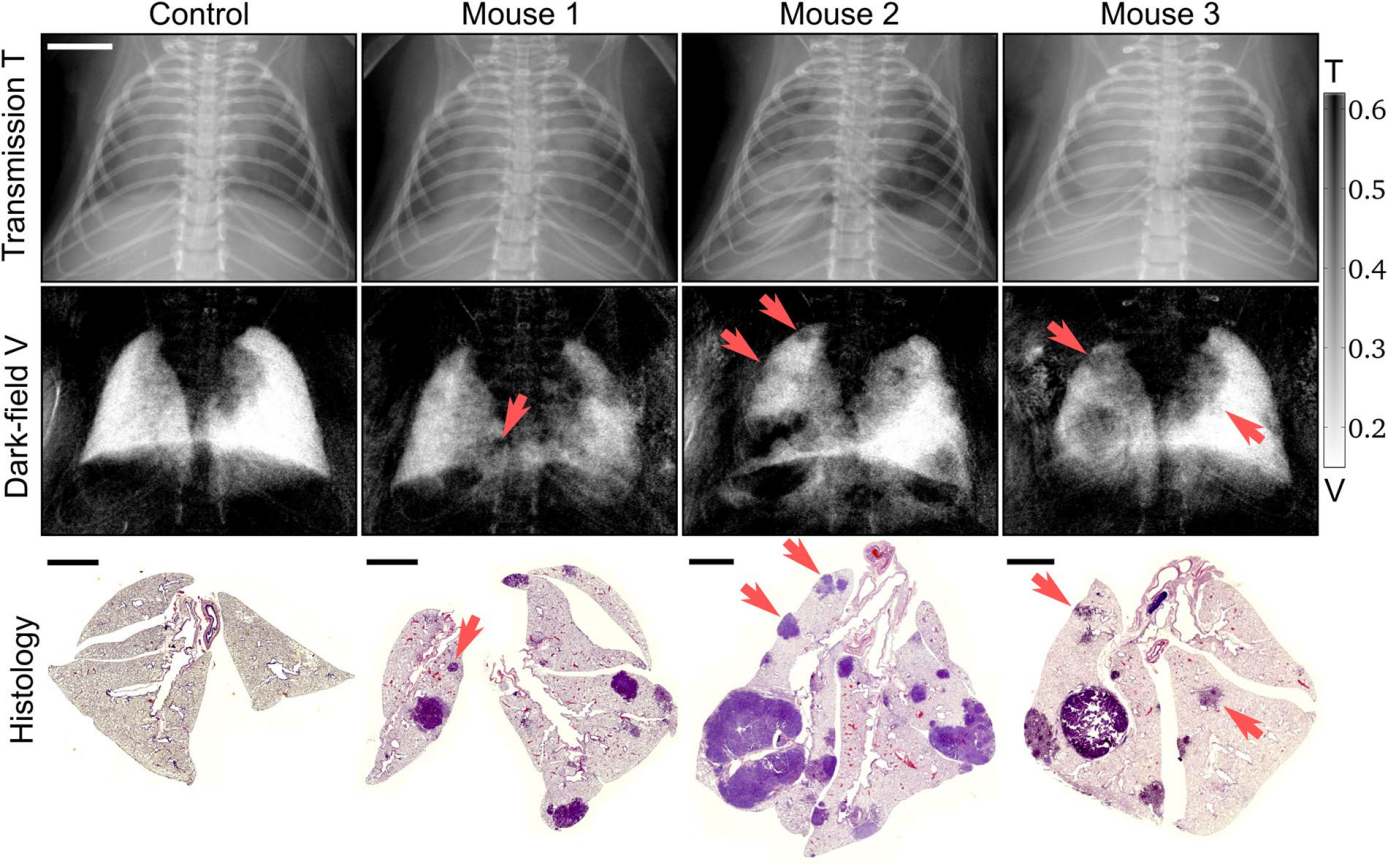
Qualitative benchmark of *in-vivo* transmission versus dark-field radiography for the assessment of lung nodules. Typical transmission and dark-field radiograms of a control mouse and three mice with lung tumors, alongside with the corresponding histological sections in H&E staining. Small nodules are much easier to detect within the dark-field image (red arrows) than in the attenuation channel, due to a minor impact of overlaying structures and the overall dominant scatter signal of the lung within the thorax. The scale bars correspond to 5 mm and 2.5 mm on the radiograms and histological sections, respectively. The color-scale corresponds to the linear signal of both dark-field and transmission images.

Eight K-ras^LA2^ mutant mice and one wildtype control animal were imaged with a dose of 1.5 mGy using the small-animal scanner (acquisition parameters in Table 1)^21^. Corresponding absorption and dark-field radiograms of three typical animals with lung tumors and one control mouse are presented in Fig. 2. In case of “Mouse 1–3”, multiple lung tumors are clearly visible in H&E staining (bottom row), proving a successful introduction of cancer in the imaged animals. Due in part to the signal from structures overlaying the lung, i.e. ribcage, heart shadow and the diaphragm, it is difficult to localize and differentiate these lung lesions on the transmission radiograms. Furthermore, small tumor lesions impose only minor variations to the overall optical density with respect to the surrounding healthy tissue, which renders an unambiguous detection of small nodules extremely challenging. In comparison, the impact of overlaying structures in dark-field imaging is much less severe, since signal arising from the lung – more precisely the alveoli – is dominant in the thorax region. This advantage enhances the assessment of lung lesions, since distortions in the homogeneous (strongly scattering) lung pattern are more easily perceived by the radiologist. Compared to the conventional transmission signal, significantly more lung tumors (especially small nodules) are detectable and visible as (poorly scattering) black voids in the dark-field projections (as indicated by red arrows). In addition, the size and location of the tumors can be more clearly identified and were found to be in agreement with the histopathological work-up.

**Table 1:** Small-animal CT scanner acquisition parameters used for the *in-vivo* X-ray dark-field radiography lung cancer study.

Source Voltage	Tube Current	Number of steps	Exposure/step	Total exposure	Scan time	Est. dose
35 kVp	0.57 mA	5	5 sec.	25 sec.	35 sec.	1.5 mGy

### Reader study: *in-vivo* dark-field radiography

In order to quantify the increase in diagnostic performance gained through X-ray dark-field imaging, a reader study was conducted. Three experienced radiologists were blinded and both transmission and dark-field radiograms presented separately, with the option of scaling data-sets freely (for more details see Materials & Methods). To achieve a statistical relevance a total of 16 murine lungs (8 cancer-bearing; 8 healthy - 7 of these from a previous study^17^) were considered.

**Figure 3:**
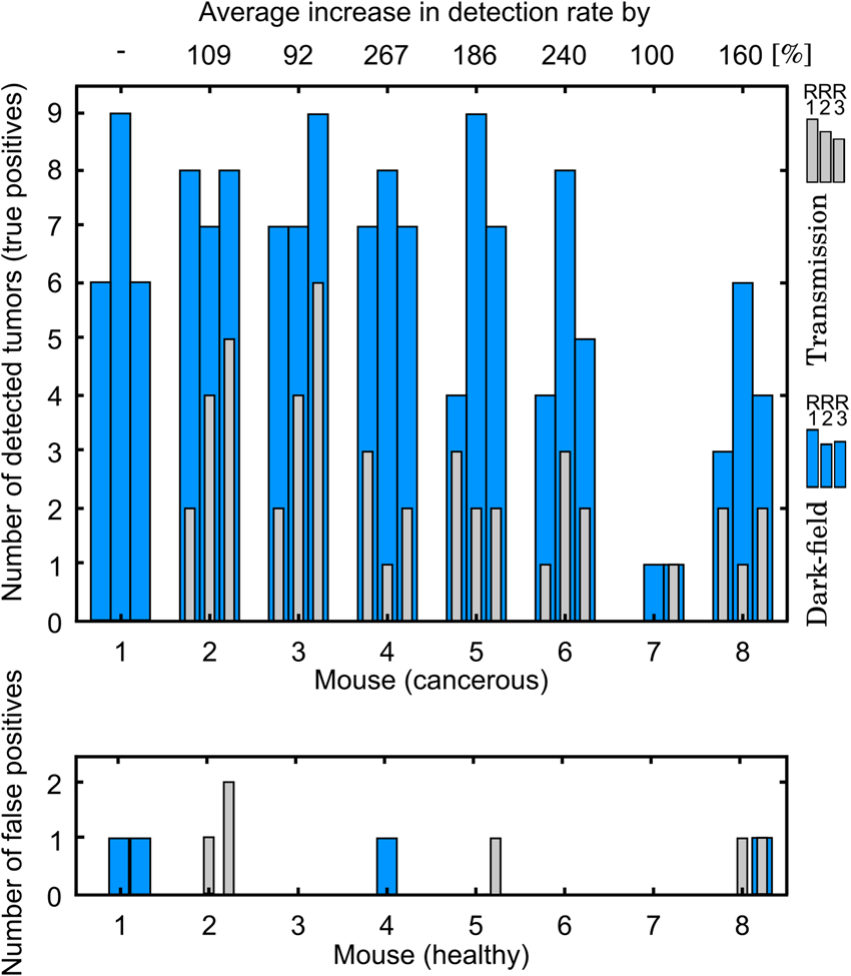
Reader study: transmission versus dark-field radiography *in-vivo*. Based on the transmission and dark-field radiograms, three experienced and blinded radiologists were asked to assess the number of radiopaque tumors in 16 mice (8 tumor-bearing, 8 healthy). A significant improvement in detection rate (true-positives) was found, when exploiting scatter-sensitive dark-field imaging, ranging from an average increase by 92% up to a maximum increase of 267%. Based on the control group our analysis provided evidence, that dark-field radiography additionally enhances diagnostic specificity, by reducing the number of false-positives from an overall of 6 to 4 incidences.

The number of detected tumors (true-positives) on transmission and dark-field images for the three readers are presented in Fig. 3, and demonstrate that dark-field imaging significantly outperforms transmission imaging with respect to the assessment of lung nodules, holding true for all three readers and each of the examined mice. We verified the here presented numbers by histology as method of reference. Note that we attribute the distinct inter-reader variance to the fact that the radiologists are trained in the evaluation of human radiographies and were not particularly trained for small-animal investigations. Compared to the transmission radiographs, we found an increase in tumor detection rates, ranging from at least 92% up to a maximum of 267%, when exploiting the dark-field channel. As a matter of fact, in the case of “Mouse 1” (see also Fig. 2) the lung was even rated as healthy on the transmission radiogram by all three radiologists, while 6, 6 and 9 small lesions were detected within the dark-field channel, respectively. Please note that we do not provide a percentage with respect to the overall number of tumors present within the lung, since an exact determination from histological work-up is ambiguous, especially in the case of very small tumors. Finally, our analysis further indicated that dark-field based diagnostics increase the diagnostic specificity of lung tumor assessment, by reducing the number of false-positives within the control group of healthy mice from an overall of 6 to 4 incidences (see also Fig. 3 - bottom).

**Figure 4:**
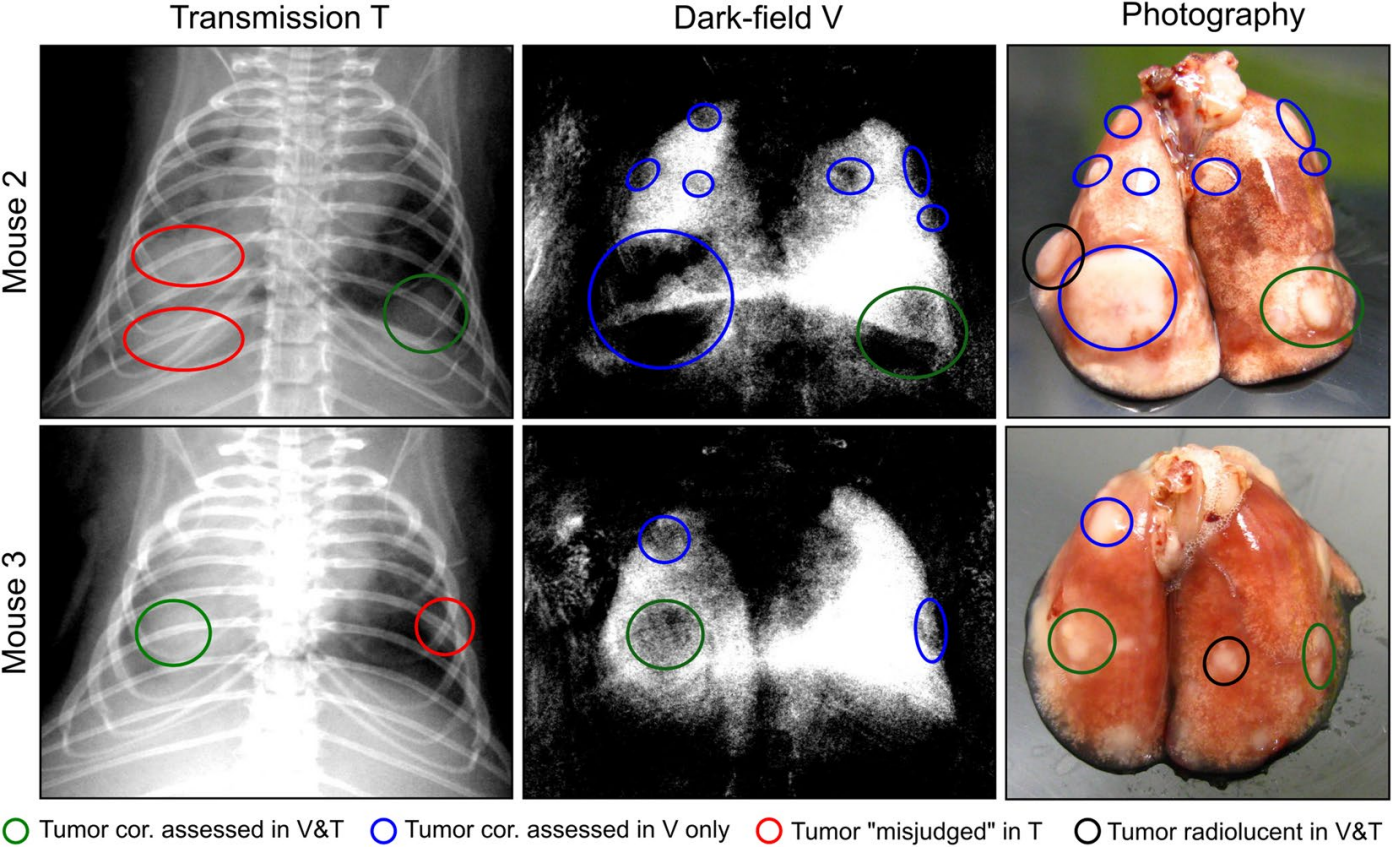
Improved assessment of tumor size, shape and delineation using X-ray dark-field radiography. Transmission and dark-field radiograms of two mice with lung cancer are taken from Fig. 2 and evaluated by a blinded reader. The green ellipses indicate tumors, which were correctly assessed in both imaging channels. The red ellipses indicate tumors which were detected in the transmission image, but misjudged with respect to size or shape, e.g. being mistakenly classified as separated (Mouse 2) or round (Mouse 3). Blue ellipses account for tumors which were correctly assessed within the dark-field radiogram only. Overlooked/missed tumors are indicated on the photograph by black ellipses.

Exceeding these insights, the reader study further indicated that scatter-based imaging enables a better determination not only of tumor numbers but also shape, size and delineation. This observation is illustrated in Fig. 4, where one of the readers digitally evaluated/sketched the extensions for each of the detected lung nodules. Photographs of the freshly excised lungs were utilized for reference. In case of “Mouse 2” for instance, two separated lesions were originally assumed, based on the transmission signal, in the bottom of the left lung. Evaluating the dark-field signal it is apparent that only one large tumor is present. In case of “Mouse 3”, the nodule in the right was estimated as round by the radiologist, while an oval tumor shape was found in the dark-field channel, in agreement with the photograph and pathology.

Nevertheless, Fig. 4 also gives evidence that dark-field radiography is not yet capable of resolving all tumors, as indicated by black circles in the photograph. This is due to the planar nature of a radiographic imaging mode, where very small tumors can potentially be superimposed by other large tumors or the prominent signal of healthy tissue. The largest missed tumor was estimated from histology and measured with a diameter of 0.7 mm.

Hence, in order to evaluate the potential of X-ray dark-field imaging in the assessment of superimposed or very small tumors, we are currently extending our study to tomographic investigations of living mice.

## Discussion

Within this study, we presented the very first dose-compatible *in-vivo* dark-field measurements of mice with lung cancer and found a superior diagnostic performance compared to conventional radiography. Thereby, we verified two main advantages associated with the dark-field signal: first, structures overlying the lung are weakly scattering and thereby do not influence the visibility of the lung. Second, micro-structural differences between healthy and cancerous lung tissue provide intrinsically enhanced scatter contrast.

**Figure 5:**
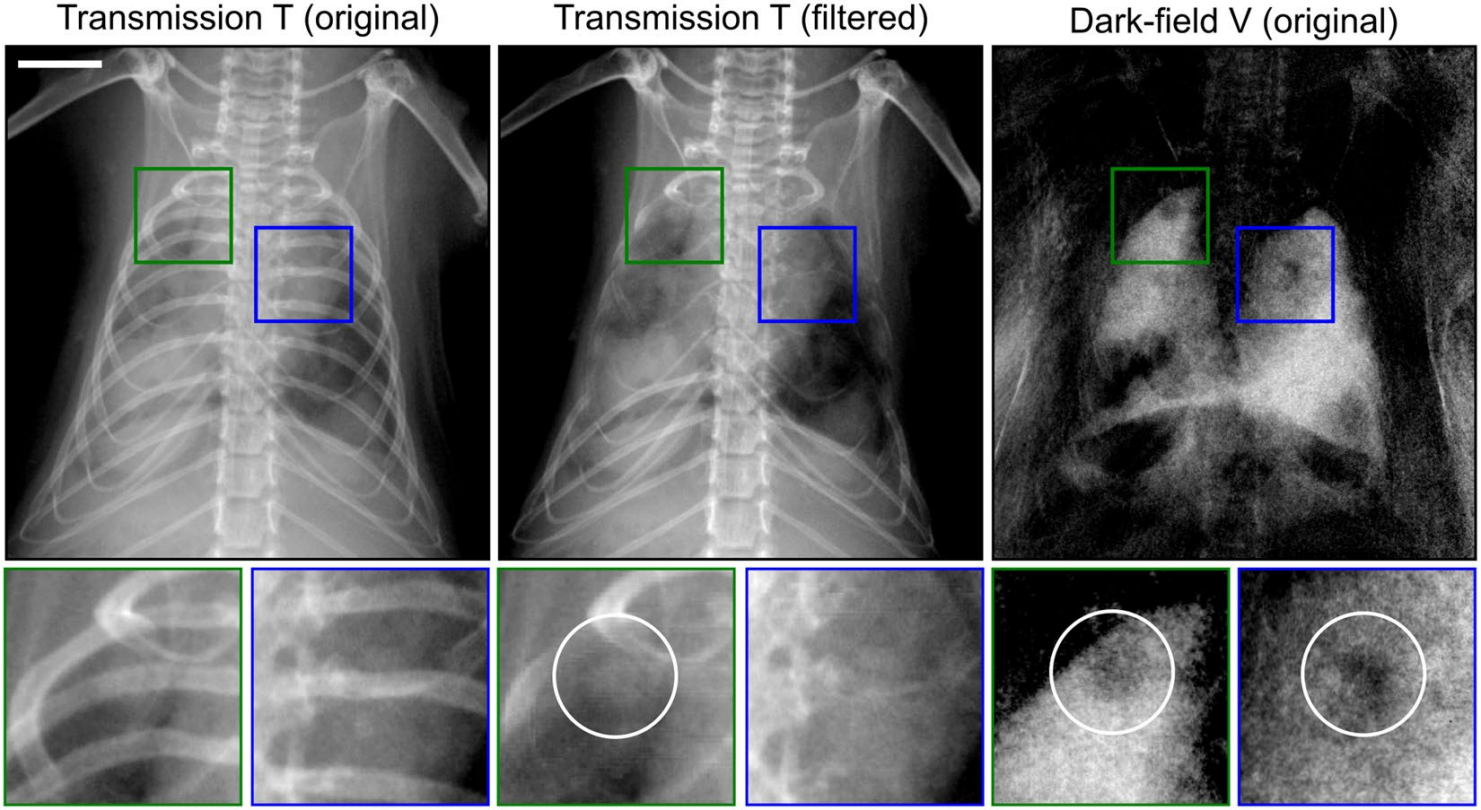
Outlook: initial comparison of ribcage-corrected transmission versus dark-field radiography and impact on tumor detection quality. In the left upper lung (green box) a small nodule is superimposed by multiple ribs and is visible only after ribcage removal, as indicated by a white circle in the filtered transmission radiogram. In case of a small lesion in the upper right (blue box), however, the tumor provides insufficient contrast with respect to healthy tissue, rather then being superimposed by bones. Consequently the suppression algorithm is not altering the detectability of the tumor. Note that both tumors are easily visible in the dark-field channel (white circles), contingent on a strongly enhanced sensitivity towards distortions in the micromorphological lung pattern caused by cancerous tissue. The scale bar corresponds to 5 mm.

While these results are very promising, we have to consider that within this proof-of-principle (reader) study none of the lately developed ribcage suppression algorithms had been applied^22–25^. Although these are yet not applied in daily clinical routine, they yield the potential to increase the quality of lung nodule visualization in transmission radiography as such^22, 25, 26^. In addition, in translating our findings to clinical imaging, it is uncertain to which degree scatter caused by the human ribcage and spine will corrupt the visibility of the human lung. Therefore, future studies will have to consider the aforementioned algorithms for transmission radiographies to provide a fair comparison. However, a very initial benchmark (Fig. 5) following the method described by von Berg *et al.* suggests that the inherent micro-structural sensitivity of dark-field imaging strongly outweighs the diagnostic benefits arising from ribcage suppression/correction algorithms only^25^: while one small nodule in the left upper lung (green box) does indeed become detectable in the transmission image after removing the ribcage, another tumor in the upper right lung (blue box) remains hidden in the transmission signal throughout rib-removal, and is reliably detected in the darkfield channel only.

Further, we hypothesize that the distinct sensitivity towards pathological changes in the pulmonary micromorphology may be of special interest for the detection of non-solid growing tumors, which are typically radiolucent in conventional radiography in early stages. Here, the tumor slowly proliferates along the alveolar walls reducing the amount of incorporated air and therewith scattering surface, while not significantly changing the optical density of related tissue^20^.

In general, X-ray dark-field imaging requires at least a doubling of dose, due to the presence of an absorber grating. We would like to point out that if a transmission image was acquired with the dose required for x-ray dark-field radiography, its SNR would have been better. However, a factor of two in dose results only in a slight increase of the SNR by a factor of $\sqrt{(2)}=1.4$. Finally, in conventional radiography systems there is an anti-scatter grid behind the sample (which also reduces the flux by approx. 20%), further reducing the difference in SNR between the attenuation images taken with a GI and the images taken with a conventional system. Therefore, we believe the presence of the GI in the imaging system does not influence the quality of the transmission images significantly. Similar imaging results have been previously reported^27^.

The mice were breathing freely during the image acquisition. As no gating was applied, this introduced some blurring to the images. However, given the typical motion amplitude of the murine lung in the range^28^ of 200 μm and the resolution of the system^29^ of approximately 70 μm, the motion artifacts have been found to be negligible in the past^30^. Therefore, the deviation of the actual tumor size from the estimated one is considered to be negligible.

Our results indicate that x-ray dark-field radiography is a promising candidate for both lung cancer screening and monitoring high-risk human patients, offering a significantly increased cancer detection rate (true-positives), without increasing the rate of false-positives. A fusion of the simultaneously acquired transmission and dark-field signal would further provide the radiologist with anatomical and scatter-sensitive (functional) imaging at a single glance. However, before this technology can be adapted to human imaging, several technical challenges have to be addressed. Experiments presented here were carried out at 35 kVp, whereas for human imaging, energies between 80–120 kVp are typically used. In general, feasibility of x-ray dark-field imaging has been demonstrated for such high energies^31–33^. These studies have also demonstrated that while Compton Scattering increases for higher x-ray energies, the G2 grating acts as an anti-scatter grating and allows thus for signal extraction. However, in general the visibility in a grating interferometer is typically reduced by a broad x-ray spectrum. Therefore, future studies should consider how the x-ray spectrum should be shaped to obtain the optimal quality for human imaging. Furthermore, the field of view has to be strongly increased. At the moment, it is limited by the size of the available gratings. Recently, several studies have reported the possibility of stitching several gratings together in order to increase the size of the field of view^34^. Tiling of the gratings as reported in the literature would allow to construct a field of view suitable for human chest imaging, and combined with a powerful enough x-ray source this would make it possible to acquire human chest radiograms with acquisition times below 1 second. Finally, the design of the grating interferometer for human imaging has to be carefully considered. On one the hand, the scattering signal obtained at e.g. 120 kVp would be significantly less than at 35 kVp, but on the other hand, the human lung is much thicker, so the signal strength should be much higher. Finally, the size of the human alveoli^35^ is approx. 200 μm, whereas those of mice are approx. 60 μm in diameter^28^. Therefore, the dark-field signal from human lungs will be weaker as described in theoretical studies^36–38^. All these effects have to be carefully considered for the design of an interferometer for human imaging. However, the sensitivity of the interferometer can be adjusted by adapting the grating periods and grating shape^36–41^. In general, for human lungs we can expect an increase in the alveoli size by a factor of approximately 3–4, which requires a smaller grating pitch by a factor of 3–4 to obtain the same signal strength for the object of the same sample thickness. However, a human lung is thicker than a murine one approximately by a factor of 10. Therefore, even if the mean energy is increased by a factor of 2, it can be assumed that the grating periods can be slightly larger for imaging of human lungs causing a slight increase in the system length. A total length of approximately 2 meters seems reasonable, which corresponds to the length of the typical system currently used for clinical radiography. To further investigate this issue, we are currently extending our investigations to larger animals, in order to model and estimate the performance of dark-field imaging for humans and disprove concerns with respect to beam hardening or patient variability.

The proposed technique is already a valuable tool for validation of novel therapeutic strategies, allowing for non-invasive monitoring in (dose-sensitive) small-animal lung cancer experiments. Currently, only micro-CT is utilized to this end detect lung tumors in mice, applying doses of 170–280 mGy per scan^7^. Here, concerns arise with respect to adverse radiation-induced side effects which may falsify the outcome of long-term studies^7^. Conventional x-ray radiography has not been used in small-animal experiments due to the lack in sensitivity. Based on our data, x-ray radiography could represent a superior imaging modality. Compared to the conventional radiography, x-ray radiography has a much higher sensitivity, and compared to the conventional micro-CT, it requires significantly less radiation dose at the cost of volumetric information.

As the proof of concept data presented here are merely qualitative, future work should focus on quantitative analysis of these findings. Dark-field CT can be employed to allow for quantitative comparison of transmission versus dark-field signal to validate the superiority of x-ray dark-field imaging with respect to the conventional radiography^42^. These are the first proof of concept findings on mice with heterogeneous tumor load and further studies have to be carried out in order to validate the minimum size of the nodules that can be reliably detected with x-ray dark-field radiography.

## Materials and Methods

### Ethics statement

Animal experiments were performed with permission of the Institutional Animal Care and Use Committee of the Helmholtz Zentrum Munich and carried out in accordance with national (Gesellschaft für Versuchstierkunde - Society for Laboratory Animal Science) and international (Federation for Laboratory Animal Science Associations) animal welfare guidelines. All experimental protocols were approved by the Institutional Animal Care and Use Committee of the Helmholtz Zentrum Munich.

### Small-animal lung cancer model and animal handling

For this study, 129 S/Sv-Kras^tm3Tyj^/J (K-ras^LA2^) mutant mice (Jackson Laboratory, Maine, USA) were used. In this mouse model, somatic activation of a latent K-ras allele with a G12D mutation at codon 12 leads to spontaneous development of early onset lung adenomas with 100% penetrance, that eventually evolve to high-grade adenocarcinomas^43^. K-ras mutation is predominantly seen in patients with non-small-cell lung cancer accounting for 20–30% of all incidences^44^.

Mice were anesthetized using intraperitoneal injection of medetomidine (500 μg/kg), midazolam (5 mg/kg), and fentanyl (50 μg/kg) during imaging and sacrificed after imaging with an overdose of ketamine and xylazin hydrochloride solution (bela-pharm, Germany). The mice were breathing freely during the image acquisition.

### Small-animal scanner

The imaging was performed using a small-animal scanner^21, 45^. The design of the scanner was optimized for murine lung imaging in a number of steps. After initial simulations^46^, the signal of the *ex vivo* lung was validated^11, 12^ before the interferometer was used to image an *in vivo* mouse^47^. Subsequently, the signal decay was validated in good agreement with simulations and micro-bubbles^10, 13^ and mouse models of emphysematous^15, 16^ and fibrotic tissue^17^. In addition to the conventional X-ray gantry, consisting of an X-ray source (fixed anode tungsten-target MCBM 65B-50 W tube (RTW, Neuenhagen, Germany)) and a flatpanel (C9312SK-06 Hamamatsu (Hamamatsu, Japan)) detector with a GOS scintillator and 50 μm pixel size, a three grating Talbot-Lau interferometer was introduced. The interferometer consists of a source grating (G0) with a period of 10 μm and gold height of 35 μm, a binary phase grating (G1) that has a period of 3.24 μm and a nickel height of 4 μm and, finally, an analyzer grating with a period of 4.8 μm and 45 μm high gold bars. The design energy of the system is 23 keV. The distances between the source, the phase grating and the analyzer grating are 30 and 14.5 cm respectively, and the interferometer is operated in the first fractional Talbot order. A piezo positioner is used to move the source grating in order to acquire the phase stepping curve^48^. A sample bed is used to position the sample right in front of the phase grating. The scanner is equipped with breath and heart rate monitoring system and a warm air fan to keep the animals temperature constant during *in vivo* imaging. Besides the attenuation and dark-field images, the scanner also provides the differential phase contrast images. However, these images are not included in this study and are only presented in supplementary Figure 1 for the interested reader.

### Reader-study

A blinded reader study was performed with three radiologists (four years of professional experience), who were familiar with the concept of X-ray dark-field imaging. The evaluation of the randomized transmission and dark-field images was performed separately with a certain temporal interval so that a recall bias was avoided, e.g. the readers could not build on the previous results. All readers were allowed to freely adjust the windowing and the magnification of the images using OsiriX Dicom Viewer (Pixmeo Sarl, Bernex, Switzerland). The readers were asked to mark lung cancer and delineate the boundaries of lung nodules.

### Histological preparations

Following intubation of the mice and diaphragm dissection, airways were inflated with 4% (w/v) paraformaldehyde (PFA). Later, the tracheae were knotted, the lungs were excised and transferred into 4% PFA-loaded tubes for tissue fixation overnight at 46 °C. After tissue processing and paraffin embedding, 3 μm thick sections were cut and placed on Superfrost^TM^ Plus Adhesion Slides (Thermo Scientific). The de-paraffinized sections were stained with hematoxylin and eosin (Carl Roth, Germany) subsequently, and dehydrated respectively in consecutively grading ethanol and xylene solutions (AppliChem, Germany). Dried sections were mounted with entellan (Merck, Germany).

## Electronic supplementary material

Supplementary phase images

## References

[1] Ferlay J (2010). Estimates of worldwide burden of cancer in 2008: Globocan 2008. International Journal of Cancer.

[2] Organization, W. H. World cancer report. Tech. Rep., World Health Organization (2014). ISBN 9283204298 2014.

[3] Herbst RS, Heymach JV, Lippman SM (2008). Lung cancer. N. Engl. J. Med..

[4] Freedman MT, Lo S-CB, Seibel JC, Bromley CM (2011). Lung nodules: Improved detection with software that suppresses the rib and clavicle on chest radiographs. Radiology.

[5] Fardanesh M, White C (2012). Missed lung cancer on chest radiography and computed tomography. Seminars in Ultrasound, CT and MRI.

[6] Oken MM (2011). Screening by chest radiograph and lung cancer mortality: the prostate, lung, colorectal, and ovarian (plco) randomized trial. JAMA.

[7] Rodt T (2011). Phantom and cadaver measurements of dose and dose distribution in micro-CT of the chest in mice. Acta radiologica.

[8] Pfeiffer F, Weitkamp T, Bunk O, David C (2006). Phase retrieval and differential phase-contrast imaging with low-brilliance x-ray sources. Nature Physics.

[9] Pfeiffer F (2008). Hard-x-ray dark-field imaging using a grating interferometer. Nature Materials.

[10] Malecki A (2014). Correlation of x-ray dark-field radiography to mechanical sample properties. Microscopy and Microanalysis.

[11] Schleede S (2012). Emphysema diagnosis using x-ray dark-field imaging at a laser-driven compact synchrotron light source. Proceedings of the National Academy of Sciences.

[12] Yaroshenko A (2013). Pulmonary emphysema diagnosis with a preclinical small-animal x-ray dark-field scatter-contrast scanner. Radiology.

[13] Velroyen A (2013). Microbubbles as a scattering contrast agent for grating-based x-ray dark-field imaging. Physics in Medicine and Biology.

[14] Velroyen A (2015). *Ex vivo* perfusion-simulation measurements of microbubbles as a scattering contrast agent for grating-based x-ray dark-field imaging. PloS one.

[15] Meinel FG (2014). Improved diagnosis of pulmonary emphysema using *in vivo* dark-field radiography. Investigative radiology.

[16] Hellbach, K. *et al.**In vivo* dark-field radiography for early diagnosis and staging of pulmonary emphysema. *Investigative radiology* (2015). 10.1097/RLI.0000000000000147 25761095

[17] Yaroshenko A (2015). Improved *In vivo* Assessment of Pulmonary Fibrosis in Mice using X-Ray Dark-Field Radiography. Sci Rep.

[18] Hellbach K (2016). Facilitated diagnosis of pneumothoraces in newborn mice using x-ray dark-field radiography. Investigative radiology.

[19] Yaroshenko A (2016). Visualization of neonatal lung injury associated with mechanical ventilation using x-ray dark-field radiography. Scientific reports.

[20] Zhang L (2012). Internal Growth of Nonsolid Lung Nodules: Radiologic-Pathologic Correlation. Radiology.

[21] Tapfer A (2012). Experimental results from a preclinical x-ray phase-contrast ct scanner. Proceedings of the National Academy of Sciences.

[22] Oda S (2009). Performance of radiologists in detection of small pulmonary nodules on chest radiographs: Effect of rib suppression with a massive-training artificial neural network. American Journal of Roentgenology.

[23] Li F (2011). Improved detection of subtle lung nodules by use of chest radiographs with bone suppression imaging: receiver operating characteristic analysis with and without localization. American Journal of Roentgenology.

[24] Ahmed, B. *et al.* Rib suppression for enhancing frontal chest radiographs using independent component analysis. In Beliczynski, B., Dzielinski, A., Iwanowski, M. & Ribeiro, B. (eds) *Adaptive and Natural Computing Algorithms*, vol. 4432 of *Lecture Notes in Computer Science*, 300–308 (Springer Berlin Heidelberg, 2007).

[25] von Berg J (2016). A novel bone suppression method that improves lung nodule detection: Suppressing dedicated bone shadows in radiographs while preserving the remaining signal. International journal of computer assisted radiology and surgery.

[26] Kakeda S (2004). Improved detection of lung nodules on chest radiographs using a commercial computer-aided diagnosis system. American Journal of Roentgenology.

[27] Scherer K (2015). Toward clinically compatible phase-contrast mammography. PloS one.

[28] Chang S (2015). Synchrotron x-ray imaging of pulmonary alveoli in respiration in live intact mice. Scientific Reports.

[29] Mueller M (2015). Contrast-to-noise ratio optimization for a prototype phase-contrast computed tomography scanner. Review of Scientific Instruments.

[30] Yaroshenko, A. *et al.* Small-animal dark-field radiography for pulmonary emphysema evaluation. In *SPIE Medical Imaging*, 90331M–90331M (International Society for Optics and Photonics, 2014).

[31] Scherer K (2015). Non-invasive differentiation of kidney stone types using X-ray dark-field radiography. Scientific reports.

[32] Ruiz-Yaniz M (2015). X-ray grating interferometry at photon energies over 180 keV. Applied Physics Letters.

[33] Thüring, T., Abis, M., Wang, Z., David, C. & Stampanoni, M. X-ray phase-contrast imaging at 100 kev on a conventional source. *Sci*. *Rep*. **4** (2014). 10.1038/srep05198 PMC4047533 24903579

[34] Meiser J (2016). Increasing the field of view in grating based x-ray phase contrast imaging using stitched gratings. Journal of X-ray science and technology.

[35] Ochs M (2004). The number of alveoli in the human lung. Am J Respir Crit Care Med.

[36] Yashiro W, Terui Y, Kawabata K, Momose A (2010). On the origin of visibility contrast in x-ray talbot interferometry. Optics express.

[37] Lynch SK (2011). Interpretation of dark-field contrast and particle-size selectivity in grating interferometers. Applied optics.

[38] Prade F, Yaroshenko A, Herzen J, Pfeiffer F (2015). Short-range order in mesoscale systems probed by x-ray grating interferometry. EPL.

[39] Yashiro W, Takeda Y, Momose A (2008). Efficiency of capturing a phase image using cone-beam x-ray talbot interferometry. Journal of the Optical Society of America. A, Optics, image science, and vision.

[40] Gromann LB (2016). Low-dose, phase-contrast mammography with high signal-to-noise ratio. Biomedical optics express.

[41] Yaroshenko A (2014). Non-binary phase gratings for x-ray imaging with a compact talbot interferometer. Optics express.

[42] Velroyen A (2015). Grating-based x-ray dark-field computed tomography of living mice. EBioMedicine.

[43] Johnson L (2001). Somatic activation of the k-ras oncogene causes early onset lung cancer in mice. Nature.

[44] D’Arcangelo M, Cappuzzo F (2012). K-Ras Mutations in Non-Small-Cell Lung Cancer: Prognostic and Predictive Value, K-Ras Mutations in Non-Small-Cell Lung Cancer: Prognostic and Predictive Value. International Scholarly Research Notices, International Scholarly Research Notices.

[45] Tapfer A (2011). Development of a prototype gantry system for preclinical x-ray phase-contrast computed tomography. Medical Physics.

[46] Malecki A, Potdevin G, Pfeiffer F (2012). Quantitative wave-optical numerical analysis of the dark-field signal in grating-based x-ray interferometry. EPL (Europhysics Letters).

[47] Bech, M. *et al.**In-vivo* dark-field and phase-contrast x-ray imaging. *Sci*. *Rep*. **3** (2013). 10.1038/srep03209 PMC3826096 24220606

[48] Weitkamp T (2005). X-ray phase imaging with a grating interferometer. Optics Express.

