# Supplementary material for: X-ray Dark-field Radiography - In-Vivo Diagnosis of Lung Cancer in Mice
Source: Sci Rep. 2017 Mar 24;7:402. doi: 10.1038/s41598-017-00489-x (PMC5428469; doi:10.1038/s41598-017-00489-x)
Supplement: Supplementary file 1 — Supplementary phase images [file 41598_2017_489_MOESM1_ESM.pdf]

X-ray Dark-field Radiography - Improved *In-Vivo* Diagnosis of Lung Cancer in Mice

Kai Scherer[1,+,*], Andre Yaroshenko[1,2,+], Deniz Ali Bölükbas[3,+], Lukas B. Gromann[1], Katharina Hellbach[4], Felix G. Meinel[4], Margarita Braunagel[4], Jens von Berg[5], Oliver Eickelberg[3], Maximilian F. Reiser[4], Franz Pfeiffer[1], Silke Meiners[3], and Julia Herzen[1]

[1]Lehrstuhl für Biomedizinische Physik, Physik-Department & Institut für Medizintechnik, Technische Universität München, 85748 Garching, Germany
[2]Philips Medical Systems DMC GmbH, 22335 Hamburg, 22335 Hamburg, Germany
[3]Comprehensive Pneumology Center (CPC), University Hospital, Ludwig-Maximilians University, Helmholtz Zentrum München, 81377 Munich, Member of the German Center for Lung Research (DZL), Germany
[4]Institute for Clinical Radiology, Ludwig-Maximilians-University Hospital Munich, 81377 Munich, Germany
[5]Philips Research Laboratories, Philips Medical Systems, 22335 Hamburg, Germany
[*]kai.scherer@tum.de
[+]these authors contributed equally to this work

Additional Information

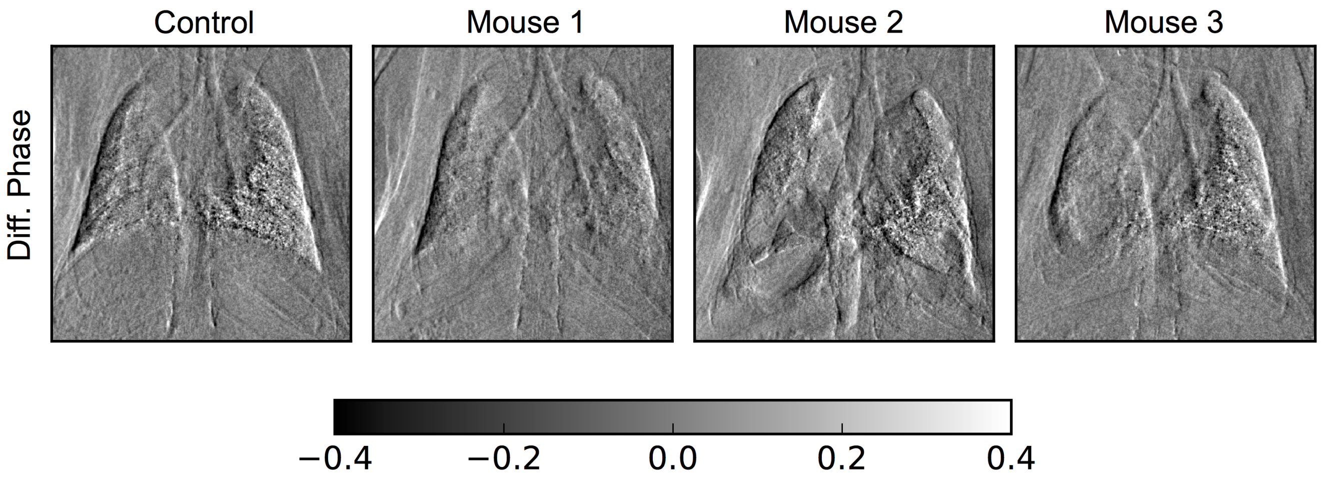

Suplementary Figure 1. Diff. phase contrast images of the mice shown in Fig. 2. For sake of completeness, this figure shows the differential phase contrast images of the same mice as shown in Fig. 2. The colorbar is given in radian. Due to the strong dark-field signal there is a lot of noise present in the region of the lung. However, this data was not included in the analysis, as we focused on the dark-field.
